# c-MYC and SIRT1 locked in a vicious cycle

**DOI:** 10.18632/oncotarget.440

**Published:** 2012-02-07

**Authors:** Antje Menssen, Heiko Hermeking

**Affiliations:** ^1^ Experimental and Molecular Pathology, Institute of Pathology, Ludwig-Maximilians-University Munich, Germany; ^2^ Experimental and Molecular Pathology, Institute of Pathology, Ludwig-Maximilians-University Munich, Germany

The c-MYC proto-oncogene represents a nodal point of gene regulation, as it integrates numerous mitogenic incoming signals and converts them into enhanced synthesis of the c-MYC transcription factor, which orchestrates the expression of hundreds of genes [1]. Deregulation of c-MYC, as it is found in ~50% of all human cancers, not only induces cellular proliferation and growth but also leads to activation of the p53 tumor suppressor protein [2], which mediates apoptosis and/or senescence. This intrinsic fail-safe mechanism prevents expansion of cells harboring dangerous oncogenic mutations. The necessity to bypass this barrier is thought to explain why p53 is frequently mutated in human cancers of all types. However, ectopic expression of c-MYC may immortalize certain primary cells in the presence of wild-type p53, suggesting that c-MYC circumvents the action of p53 by alternative mechanisms. We hypothesized that c-MYC may activate a factor, which abrogates or down-regulates p53 function directly. An attractive candidate for this function was SIRT1, an NAD^+^-dependent deacetylase, which has been shown to inhibit the function of p53 [3]. Recently, four papers showed that c-MYC and SIRT1 regulate each other via feedback loops [4-7]. The three more recent publications suggest the existence of a positive feedback loop between c-MYC and SIRT1 [4-6], whereas a study published earlier entertains a negative feedback [7]. The positive feedback suggests an oncogenic role of SIRT1, whereas the negative feedback implies a tumor suppressive activity of SIRT1. In our hands activation of conditional c-MYC alleles resulted in a robust increase in SIRT1 protein levels and activity. This was mediated on one hand via the direct induction of the *NAMPT* gene, which encodes nicotinamide phosphoribosyltransferase, the rate limiting enzyme of the NAD^+^ salvage pathway. The resulting increased NAD^+^ levels are known to mediate the activation of SIRT1 [8]. A second mode of SIRT1 activation was through sequestration of the SIRT1 inhibitor DBC1 (deleted in breast cancer 1) by c-MYC. In c-MYC-immortalized cells the increased levels and enzymatic activity of SIRT1 were necessary to prevent senescence and c-MYC-induced apoptosis. Furthermore, c-MYC is bound and deacetylated by SIRT1 which resulted in reduced K48- and increased K63-ubiquitin linkage, and ultimately increased c-MYC stability and activity. In cancer cells deregulation of this reciprocal activation between c-MYC and SIRT1 presumably contributes to evasion of senescence and apoptosis [6].

Increased expression of SIRT1 mRNA in cells with elevated c-MYC or N-MYC levels suggested that c-MYC may transactivate the SIRT1 gene [5, 7]. Although we could not detect an increase in SIRT1 mRNA after c-MYC activation [6], a concomitant induction of SIRT1 mRNA and post-transcriptional activation of the SIRT1 enzyme by c-MYC, as described above, may act synergistically. In the three studies describing a positive feedback, this involves a direct physical interaction between c-MYC and SIRT1, leading to increased c-MYC function [4-6]. Only Yuan et al. observed that the interaction with SIRT1 reduced c-MYC stability and inhibited c-MYC functions, such as transformation and transactivation [7]. The region of MYC interacting with SIRT1 was reported to be either the C-terminus of c-MYC [4] or MYC-Box I within the N-terminus of N-MYC [5]. Although three studies document deacetylation of c-MYC as a result of the SIRT1/c-MYC interaction, there are considerable discrepancies with respect to the functional consequences of c-MYC deacetylation. In agreement with our own observations obtained for c-MYC, Marshall et al. report that the N-MYC protein is stabilized in the presence of SIRT1. However, this is mediated indirectly through a novel transcriptional repressor complex consisting of N-MYC and SIRT1, which down-regulates expression of mitogen-activated protein kinase phosphatase 3 (MKP3) and thereby increases ERK-mediated phosphorylation of N-MYC at serine 62. Mao et al. did not observe significant effects of SIRT1 on c-MYC protein levels. Furthermore, they found that SIRT1 binds to the C-terminus of c-MYC, which mediates heterodimerization with MAX. Interestingly, SIRT1 expression increased the c-MYC/MAX association and also enhanced c-MYC transcriptional activity. Taken together, the majority of the reported analyses (3 of 4) describe a positive feedback between c-MYC and SIRT1. Multiple mechanisms of c-MYC activation seem to be involved in this feedback. Indeed, the SIRT1-mediated increase in c-MYC expression and function by enhanced ubiquitin 63 linkage, serine 62 phosphorylation and c-MYC/MAX heterodimerization may occur in parallel and act synergistically. In conjunction with the inactivation of p53 and/or other tumor suppressive SIRT1 substrates, SIRT1 therefore seems to act pro-tumorigenic at least in the context of cells and tumors, which display alterations in c-MYC-activating pathways or c-MYC itself.
